# Healthy lifestyle promotion via digital self-help for mental health patients in primary care: a pilot study including an embedded randomized recruitment trial

**DOI:** 10.1017/S146342362300049X

**Published:** 2023-09-20

**Authors:** Karoline Kolaas, Erland Axelsson, Erik Hedman-Lagerlöf, Anne H. Berman

**Affiliations:** 1 Centre for Psychiatry Research, Department of Clinical Neuroscience, Karolinska Institutet, Stockholm, Sweden; 2 Liljeholmen Academic Primary Care Clinic, Stockholm, Sweden; 3 Division of Psychology, Department of Clinical Neuroscience, Karolinska Institutet, Stockholm, Sweden; 4 Gustavsberg Academic Primary Care Clinic, Gustavsberg, Sweden; 5 Division of Clinical Psychology, Department of Psychology, Uppsala University, Uppsala, Sweden

**Keywords:** common mental disorders, digital support, lifestyle behaviors, lifestyle promotion, primary health care, web-based intervention

## Abstract

**Aim:**

This study piloted a digital self-help intervention facilitating healthy lifestyle for patients with mental health problems in primary care.

**Background:**

Patients with mental health problems show more unhealthy lifestyle behaviors than the general population and prior research indicates that healthy lifestyle behaviors can improve mental health.

**Methods:**

This pilot study assessed use of a self-help digital intervention for healthy lifestyle promotion and included an embedded randomized recruitment trial, where all patients were randomized to digital self-help plus treatment as usual (TAU) or to TAU only. Patients seeking help for mental health problems were recruited from two primary care clinics in Stockholm, Sweden, and offered participation in a healthy lifestyle promotion study via digital self-help. Outcome measures included use-related assessment of inclusion and follow-up rates at both clinics, participant characteristics, and intervention adherence. Secondary outcomes included depression (the Patient Health Questionnaire-9) and anxiety (the GAD-7) up to 10 weeks, and changes in alcohol and tobacco use, physical activity, and diet.

**Results:**

The study included 152 patients. The recruitment rate, initially low, increased after involving the clinicians more and maintaining more frequent contact with the patients. The 10-week missing data rate was 33/152 (22%). Participants were 70% (106/152) women, with a mean age of 42 years (SD = 14); fewer than half (38%, *n* = 58/152) had one or more high-risk unhealthy behaviors at inclusion. Psychiatric symptoms were moderate at baseline and declined in both groups after 10 weeks (d = 0.57–0.75). No between-group effects over time occurred on depression (b = 0.3 [95% CI −1.6, 2.2]; d = 0.06), anxiety (b = −0.7 [−2.5, 1.2]; d = 0.13), or lifestyle behaviors (b = 0.01 [−0.3, 0,3]; d = −0.01).

**Conclusions:**

Recruitment routines seemed to be decisive for reaching as many patients as possible. The relatively low rate of unhealthy lifestyle behaviors and small effect sizes suggests that the intervention may only suit patients at risk.

**Trial registration:**

ClinicalTrials.gov NCT03691116 (01/10/2018), focusing on the embedded trial. Retrospectively registered for the first clinic and prospectively for the second clinic.

## Background

Lifestyle-related non-communicable diseases such as coronary heart disease and type 2 diabetes are the primary cause of death worldwide and constitute one of the largest challenges in health care today (GBD, 2018). Unhealthy behaviors that predict non-communicable diseases include smoking, alcohol consumption, insufficient physical activity, and unhealthy dietary habits. Patients with mental health problems show a larger burden of unhealthy lifestyle behaviors than the population in general (Strine *et al.*, 2008; de Wit *et al.*, 2010). A relationship has been found between better mental health and higher frequency of physical activity, moderate alcohol intake, nonsmoking, and a healthy weight (Velten *et al.*, 2014). An increasing body of research indicates that healthy lifestyle behaviors can improve mental health (Firth *et al.*, 2020). For example, there is growing evidence for smoking as a possible causal factor for depression, anxiety disorders, and insomnia (Firth *et al.*, 2020). Alcohol consumption contributes to the risk of depression (Boden and Fergusson, 2011), increased physical activity to reducing anxiety (Wegner *et al.*, 2014; Takacs and Stauder, 2016), as well as affecting mild to moderate depression at the same magnitude as anti-depressive medication or cognitive behavioral therapy (Hallgren *et al.*, 2016; Netz, 2017).

Patients with common mental disorders, such as mild to moderate depression and anxiety, are increasingly being treated at primary care health clinics, rather than being referred for specialist psychiatric treatment (Mancini, 2021). Given the links between mental health and lifestyle behaviors, this means that lifestyle behaviors can and should be addressed for this group of patients. Promotion of healthy lifestyle behaviors is generally recommended for primary care patients in clinical guidelines (WHO, 2012), and there is a need to expand availability of healthy lifestyle promotion to improve mental health (WHO, 2021). The absence of structured methods of implementation tends to be frustrating for health professionals and leaves them to individually decide when and how to promote lifestyle habits (Berman *et al.*, 2018). One potential method for lowering the threshold for implementation and dissemination is to offer digital interventions, which have potential to improve diet, physical activity, and tobacco and alcohol use (Afshin *et al.*, 2016). Digital interventions can be delivered in a general non-personal way, which can reduce patientʼs feelings of being judged (Albury *et al.*, 2019). However, some studies have indicated that less is known about the effects on populations with a lower socioeconomic status (Western *et al.*, 2021).

The implementation of structured lifestyle promotion in primary care has been studied in Sweden, using a brief pencil-and-paper intervention, the ‘Health Profile’ (HP) (Blomstrand *et al.*, 2005). The health status of a person’s lifestyle can be measured using biological risk markers, as well as behaviors. The HP measures behaviors to identify risk as well as offer an intervention, based on prior research indicating that prevention should begin early to reduce the risk of cardiovascular disease (Persson *et al.*, 1998) as well as other non-communicable diseases, including mental health. The intervention consists of self-monitoring as well as a personal counseling session to motivate patients to change unhealthy behaviors. The HP includes questions about four lifestyle behaviors (smoking, alcohol, diet, and physical activity) as well as sleeping habits, stress, and general life situations. The results yield an overview of risk factors, and the risk areas are classified as ‘’good’ (green), ‘less good’ (orange), or ‘risk’ (red), illustrated in a color health profile. Following generation of the personal health profile, the intervention offers information and brief evidence-based advice on different ways to change unhealthy behaviors. The HP counseling session targets patients with unhealthy behaviors and is based on motivational interviewing (Berman *et al.*, 2020). The intervention is described in more detail elsewhere (Persson *et al.*, 1998; Blomstrand *et al.*, 2005).

Observational results from a naturalistic study showed that the HP attracted primary care patients with unhealthy lifestyle behaviors, and yielded significant within-group reductions in body mass index, waist circumference, waist-hip ratio, blood pressure, and p-glucose observed at 1-year follow-up (Blomstrand *et al.*, 2012). The intervention has also shown significant associations with healthy lifestyle changes among patients from socioeconomically vulnerable groups as well as those from higher socioeconomic levels (Waller *et al.*, 2016). However, the intervention has not been previously tested in a digital format and has not been evaluated in a randomized controlled study. Also, little is known about the utility of such interventions specifically targeting primary care patients seeking help for mental health problems. In sum, more knowledge is needed about lifestyle interventions in digital format, to promote healthy behaviors for primary care patients, both those presenting with somatic problems as well as those with mental health problems. Also, studies are needed to verify the use of technology and to explore ways to change unhealthy behaviors in patients with mental illness in primary health care.

### Aim of this study

The main aim of this study was to evaluate the use of healthy lifestyle promotion via digital intervention for patients seeking mental health services in primary care. An additional aim was to inform the suitability of executing a larger multi-center randomized controlled trial (RCT) to evaluate the intervention. The intervention had neither previously been assessed in digital format, nor been offered to the target group in this study, so the investigation was exploratory, without a priori, directional hypotheses.

The primary study aim was thus to evaluate the use of a digitalized version of the paper-and-pencil HP intervention described above (Blomstrand *et al.*, 2005). The specific research objectives included: a) to investigate what proportion of the estimated number of patients eligible for the study would participate, and how recruitment might differ in two variations of procedural design; b) to explore patient characteristics, including to what extent primary care patients seeking help for mental health problems show unhealthy lifestyle behaviors; c) to assess intervention adherence, in terms of the proportion of patients logging in to the digital intervention at least once; as well as to assess how large a proportion of patients would opt into a motivational interviewing (MI) counseling session when it was available.

The secondary aim was, in turn, to evaluate the randomization process, in preparation for the planned RCT; an embedded recruitment trial design was chosen to fulfill this aim. Specific research objectives included comparing follow-up rates by group and, as a secondary objective, analyzing any changes occurring in depression, anxiety, and lifestyle behaviors.

## Materials and methods

### Design and setting

This was a pilot study that evaluated a digital self-help version of the HP, an intervention for healthy lifestyle promotion among primary care patients (Blomstrand *et al.*, 2005; Blomstrand *et al.*, 2012). The target population was patients seeking help in primary care for mental health problems. An embedded recruitment trial evaluated the randomization and analysis procedures, based on Study within a Trial (SWAT) methodology (Treweek *et al.*, 2018). In view of the study’s clinical setting, a pragmatic RCT design was used (Ford and Norrie, 2016). About one-third of the way through the study, slow participant recruitment motivated a move of the trial from the initial primary care clinic to a second clinic. This move coincided with change of employment for the first author, who went from a position as treating psychologist at the first clinic to managing the psychosocial unit at the second clinic. The trial was thus carried out in two phases. The first phase took place in 2017–2018 at the Gustavsberg Primary Care Clinic (clinic A), situated 25 min outside of Stockholm, Sweden. The second phase took place in 2019 at the Liljeholmen Primary Care Clinic (clinic B), a more central location in Stockholm. Both clinics serve over 30 000 patients each and include patient groups in catchment areas with better overall health, compared to the general Swedish population (Makenzius, 2019); the patient groups also have a higher income than the national Swedish average (Statistics Sweden, 2021). The inclusion plan aimed at 150 participating patients, based on clinical judgment of the number needed to assess the distribution of unhealthy lifestyle behaviors in the group, to evaluate additional outcomes and to understand potential intervention effects. No power analysis for the embedded trial was conducted. The study was approved by the Regional Ethics Review Board of Stockholm (2016/1013-31/4) and was registered at ClincalTrials.gov (NCT03691116). This pre-registration primarily focused on the SWAT procedures for the embedded trial. Therefore, the content of this article deviates to some extent from the information available at ClinicalTrials.gov. The SWAT outcomes, resulting from the embedded randomized recruitment trial, generally follow the CONSORT 2010 extension for randomized pilot and feasibility trials (Eldridge *et al.*, 2016).

### Participants and recruitment

Patients referred to psychological assessment in Swedish primary care are most often individuals with mild to moderate common mental illness, usually related to anxiety, depression, or stress. At both clinics, consecutive patients referred by their general practitioner for psychological assessment within the clinic were assessed for eligibility. At clinic A, patients received information by postal mail about the study and were also given written information at the clinic reception desk before their first appointment with a mental health professional. Following the slow inclusion rate at clinic A, the project was restarted in clinic B where new routines were established to increase the recruitment rate. In clinic B, clinical staff were involved to a larger extent, for example, by telephoning all patients to ensure that they had received the information by postal mail and asking if they had any questions about the study. When patients did not reply, a text message reminder was sent. At clinic B, instead of receiving information at the clinic reception desk, patients were asked by their mental health professional at the first appointment if they had received information about the study. Patients at both clinics could sign their consent to participate at home and send it back in a prepaid envelope, or leave it in a sealed envelope in the reception at the clinic. In order to be included in the study, patients were required to be fluent in Swedish, have access to an electronic device capable of running a modern web browser, and have an electronic ID. In order to obtain a clinically relevant sample with high external validity, no exclusion criteria were applied, such as severe mental illness or an upper age limit. All patients received treatment as usual (TAU), described below.

### Randomization

**Figure 1.**
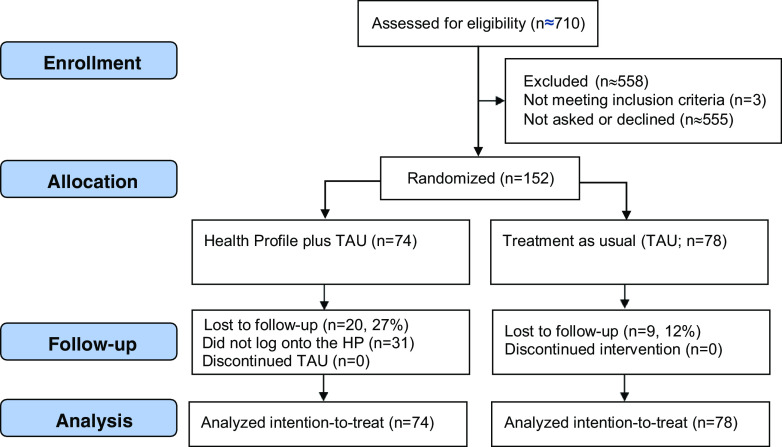
Flow diagram.

Patients were randomly allocated (1:1) using random.org to either the HP in addition to TAU group, or to a control group receiving only TAU. To reduce researcher bias, the randomization procedure was conducted by undergraduate or graduate-level psychology students who were either doing a clinical internship or were working as psychologist assistants. Participants were informed of their allocation via postal mail. Those randomized to the HP group were sent instructions about how to log on to the digital platform, with the information that they would be contacted again to complete the 10-week assessment, and those randomized to TAU were informed that they would be contacted again to complete the 10-week assessment. See Figure 1 for the trial flow, including follow-up rates.

### Interventions

#### Digital lifestyle intervention – the Health Profile (HP)

The HP consists of a self-report questionnaire followed by brief feedback and suggestions about how to change behaviors to improve one’s health. The questionnaire covers the domains of smoking, alcohol, diet, and physical activity and also mental stress and work/unemployment situation, and is described in detail elsewhere (Blomstrand *et al.*, 2005). The original pencil-and-paper health profile was converted by authors AHB and KK to a digital format in collaboration with representatives from the original research group (Blomstrand *et al.*, 2012). The HP was delivered via a publicly funded Swedish digital intervention site called the ‘Support and treatment platform’. This platform was chosen because it is used in ordinary healthcare nationwide and offers the potential for scaling up the HP for future use in clinical settings, not only in research studies. The original HP included an optional single counseling session focusing on lifestyle behavior change based on MI (Berman *et al.*, 2020). According to the original study plan, the MI intervention had been postponed to a future study, but in view of the delays in recruitment at clinic A, the decision was made to offer MI sessions at clinic B, to explore potential benefits of adding MI. The MI sessions were 30–60 min long, focused on the health behavior changes that the participant wanted to make, and were delivered by a psychology student or nurse, both with training in MI.

#### Treatment as usual (TAU)

TAU typically consisted of a structured anamnestic and diagnostic interview with feedback to the patient. After this, an individual treatment plan was developed in collaboration with the patient, based on national treatment guidelines (Swedish National Board of Health and Welfare, 2017). Treatment usually consisted of guided self-help or cognitive behavior therapy for anxiety or depression in individual or group formats. Patients who had sought help but did not fulfill the criteria for an anxiety or depression diagnosis were offered counseling based on cognitive behavioral techniques with focus on the main problem area if a patient-provider dialogue indicated that significant suffering was being subjectively experienced by the patient. If patients needed assessment for severe mental illness, such as bipolar disorder or schizophrenia, they were referred to specialized psychiatric care but were not excluded from the study.

### Outcomes

#### Measurement strategy

Outcomes consisted of the inclusion rate, the proportion of unhealthy lifestyle behaviors in the sample, adherence to the protocol (proportion of HP participants logging on at least once), and missing data. Clinical outcomes measured were depression and anxiety symptoms and lifestyle behaviors. All participants completed self-report questionnaires at baseline and 10 weeks later. In accordance with the intention-to-treat principle, all patients, including those who had dropped out of treatment or had been referred to another clinic, were encouraged to complete the 10-week assessment.

#### Measures

To measure symptoms of depression in the embedded recruitment trial, the Patient Health Questionnaire-9 (PHQ-9) was used, previously found to exhibit adequate psychometric properties (Kroenke *et al.*, 2001). The PHQ-9 was scored 0-27, <5 being indicative of minimal depression, 5–9 mild, 10–14 moderate, 15–19 moderately severe, and 20–27 severe depression. Anxiety was measured using the GAD-7, previously also found to exhibit adequate psychometric properties (Spitzer *et al.*, 2006). The GAD-7 was scored 0–21, <5 being indicative of minimal symptoms, 5–9 mild, 10–14 moderate, and 15–21 severe anxiety symptoms. Lifestyle behaviors were measured using the Lifestyle Behaviors Questionnaire (LBQ) (see Supplement 1), developed for clinical practice in the Stockholm Region based on lifestyle recommendations from the Swedish National Board of Health and Welfare. Although the LBQ, to our knowledge, has not undergone psychometric evaluation, it was used for pragmatic reasons; i.e., it was in widespread clinical use and its integration with the digital medical record system made it convenient to administer. The LBQ comprises 11 items and covers (i) tobacco use, (ii) alcohol use, (iii) insufficient physical activity, and (iv) unhealthy diet. There is no standardized way to score the LBQ. Therefore indices were calculated, corresponding to each domain of lifestyle behaviors, where patients were classified as low, moderate, and high risk for each domain. A numerical overall unhealthy lifestyle behaviors score is also reported, ranging from 0 (high risk) to 8 (low risk). The LBQ scoring procedure is described in more detail in Supplement 1, which contains detailed definitions of unhealthy lifestyle behaviors and reports the number of patients with three different levels of risk for the four behaviors covered by the LBQ.

### Statistical analysis

To evaluate secondary outcomes from the embedded randomization trial, analyses were conducted in Stata 14.2 on an intention-to-treat basis, meaning that data were retained from all participants who were included in the study, regardless of adherence, so as to maintain experimental control of the study outcomes. Multiple imputation by chained equations (20 datasets) was employed, conducted separately for each condition to preserve interactions (HP vs. TAU), and using the following predictors in addition to symptom scores as decided a priori: age, sex, participation in MI (yes/no), and clinic (A vs. B). Using the multiply imputed data, change in depression symptoms, anxiety, and the total unhealthy lifestyle behaviors score was analyzed in a linear mixed-effects regression framework. These models were fitted using maximum likelihood estimation and included a random intercept and the fixed effects of time (baseline = 0, 10 weeks = 1), group (TAU =  0, HP = 1), and the time × group interaction. Standardized effects are reported in terms of Cohen’s d. Between-group effect sizes were calculated as the time × group coefficient divided by the pooled observed endpoint standard deviation, and within-group effects were calculated as the model-implied change score divided by the observed change score’s standard deviation. For the d statistic, absolute values around 0.20 are usually regarded as small, 0.50 as moderate, and 0.80 as large (Cohen, 1988). The lifestyle domain indices (smoking, alcohol, physical activity, and diet) were not suitable for linear regression and were instead analyzed using χ^2^ tests, covering the distribution of improved, unchanged, and worsened risk behaviors (3 categories) across the HP and TAU groups (2 categories).

## Results

### Overall recruitment

Recruitment began in February 2017 and the last 10-week assessment was completed in November 2019, with a pause from spring 2018 to early 2019 due to the change of location. A total of 155 patients gave their written informed consent. Three of these patients were excluded after consenting to participate because one lacked electronic ID and two did not complete the baseline measures. The recruitment rate, i.e. the ratio of patients booked for their first appointment to the number of patients included, was approximately 10% at clinic A (44 included out of ≈440), and about 40% at clinic B (108 included out of ≈270).

### Patient characteristics

The average patient was a 42-year-old woman with moderate symptoms of depression and anxiety. Unhealthy lifestyle behaviors were reported as follows: about 5% reported daily smoking, 11% reported moderate or high risk of alcohol consumption, 26 % reported insufficient physical activity (less than 150 min/week), and 14% of the patients reported unhealthy dietary habits. In all, 38% (*n* = 58) reported at least one high-risk unhealthy lifestyle behavior, 7% (*n* = 10) reported two high-risk behaviors, and 1% (*n* = 2) reported three high-risk behaviors. None reported high-risk behaviors in all four areas. The remaining 62% (*n* = 94) reported no high-risk lifestyle behaviors. Patient baseline characteristics are presented in Table 1.

**Table 1.** Sample characteristics and primary outcomes, pre- and post-intervention

Characteristics	Health Profile (HP; *n* = 74)^a^	Treatment as usual (TAU; *n* = 78)	Total (*N* = 152)
Age, M (SD), range	41 (13)	42 (15)	42 (14)
	18–78	18- 82	18–82
Women, *n* (%)	48 (65%)	58 (74%)	106 (70%)
Study site, *n* (%)			
Clinic A	23 (52%)	21(48%)	44 (100%)
Logged on to HP at least once	7/23 (30%)		
Clinic B	51 (47%)	57 (53%)	108 (100%)
Logged on to HP at least once	36/51 (71%)		
Pre-intervention high-risk unhealthy behavior^b^	29 (39%)	29 (37%)	58 (38%)
Post-intervention high-risk unhealthy behavior^b^	14/54	16/69	30/123
	(26%)	(23%)	(24%)
Pre-intervention PHQ-9, M (SD)	11.5 (5.6)	12.1 (6.0)	11.8 (5.8)
Post-intervention PHQ-9, M (SD)	7.7 (4.7)	7.6 (5.3)	7.6 (5.0)
Pre-intervention GAD-7, M (SD)	10.4 (5.3)	10.3 (5.3)	10.3 (5.2)
Post-intervention GAD-7, M (SD)	6.9 (4.7)	7.2(5.5)	7.2 (5.1)

a The Health Profile group received the intervention as a complement to treatment as usual.

b High-risk unhealthy behavior is defined as at least one of the following: (1) daily smoking, (2) alcohol >9 W/>14 M drinks per week *and* binge drinking more than once a month, (3) physical activity <75 min/week of vigorous exercise *and* <150 min low-intensity exercise/week, and (4) diet score of 0–4 on the unhealthy diet index.

### Intervention adherence

In total, 58% (*n* = 43) of the patients randomized to the HP group logged on to the platform at least once. However, at clinic A, 30% (7/23) logged onto the platform at least once, and after modifying the inclusion routine at clinic B, 71% (36/51) of the patients logged on at least once, a significant difference (χ^2^ [2,74] = 10.5, *P* = 0.001). At the 10-week assessment, the rate of missing data was 22% (33/152). Fifteen of the 51 patients (29%) enrolled in the HP group at clinic B accepted participation in the optional MI session they were offered.

### Depression, anxiety, and lifestyle outcomes

The results from the embedded randomized trial are displayed in Table 2. Both groups showed significant moderate within-group reductions in depression and anxiety. No significant HP vs. TAU differences in depression or anxiety were found over time and the corresponding standardized effects were small ([PHQ-9]: b = 0.3 [95% CI −1.6, 2.2]; d = −0.06); ([GAD-7]: b = −0.7 [−2.5, 1.2]; d = 0.13). When separately analyzing the participants who received MI, again the between-group effects on depression and anxiety were not significant between the HP + MI group and TAU (depression [PHQ-9]: b = 2.4 (95% CI) −0.7, 5.6, *P* = 0.127; anxiety [GAD-7]: b = −0.8 (95% CI) −3.4, 1.8, *P* = 0.532. Linear mixed-effects regression analysis of the total lifestyle behavior index showed no significant between-group effects up to the 10-week assessment (b = 0.01 (95% CI) −0.3, 0.3), see Table 2.

**Table 2.** Changes in depression, anxiety, and lifestyle behaviors as modeled using linear mixed-effects regression

Outcome	Scale (range)	Group	Within-group pre-post effects		Between-group effects (time × group)		
			b (95% CI)	Cohen’s d	b (95% CI)	*p*	Cohen’s d
Depression	PHQ-9 (0–27)	HP	−3.8 (−5.3, −2.4)	0.74	0.3 (−1.6, 2.2)	0.774	−0.06
		TAU	−4.1(−5.4, −2.9)	0.75			
Anxiety	GAD-7 (0–21)	HP	−3.5 (−4.9, −2.0)	0.57	−0.7 (−2.5, 1.2)	0.488	0.13
		TAU	−2.8 (−4.0, −1.6)	0.67			
Lifestyle behaviors	LBQ (0–8)	HP	0.1 (0.1,0.3)	−0.11	0.01 (−0.3, 0,3)	0.938	−0.01
		TAU	0.1 (0.1, 0.3)	−0.10			

HP = Health Profile; LBQ = Lifestyle Behaviors Questionnaire; TAU = treatment as usual.

*Note:* For depression and anxiety, higher scores indicate a more severe problem. For lifestyle behaviors, higher scores indicate healthier behaviors; i.e., lower levels of risk.

The results of χ^2^ test analyses of changes in unhealthy behaviors showed no significant differences over time, see Table 3. Finally, there were no significant differences in unhealthy behaviors between participants who received MI compared to the rest of the HP intervention group according to the total lifestyle index score (b = 0.0 [−0.5, 0.6], *P* = 0.930).

**Table 3.** Risky lifestyle behaviors

Risk behaviors^b^	Total (*N* = 152)^a^		HP (*n* = 74)		TAU (*n* = 78)		Comparison^c^	
	Pre-intervention	Post-intervention	Pre	Post	Pre	Post	X^2 (df = 2)^	*P*-value
**Smoking**								
High risk	7 (4.6%)	6 (5.0%)	3 (4.1%)	3 (5.7%)	4 (5.2%)	3 (4.5%)	2.4794	0.289
Moderate risk	11 (7.3%)	9 (7.5%)	5 (6.8%)	4 (7.6%)	6 (7.8%)	5 (7.5%)		
Low risk	133 (88.1%)	105 (87.5%)	66 (89.2%)	46 (86.8%)	67 (87.0%)	59 (88.1%)		
**Alcohol**								
High risk	3 (1.97%)	4 (3.3%)	1 (1.4%)	2 (3.8%)	2 (2.6%)	2 (2.9%)	0.7969	0.671
Moderate risk	13 (8.6%)	7 (5.79%)	6 (8.1%)	1 (1.9%)	7 (8.9%)	6 (8.8%)		
Low risk	136 (89.5%)	110 (90.9%)	67 (90.5%)	50 (94.3%)	69 (88.5%)	60 (88.2%)		
**Physical activity**								
High risk	40 (26.3%)	24 (19.8%)	23 (31.0%)	12 (22.6%)	17 (21.8%)	12 (17.7%)	0.2388	0.887
Moderate risk	61 (40.1%)	48 (39.7%)	32 (43.2%)	23 (43.4%)	29 (37.2%)	25 (36.8%)		
Low risk	51 (33.6%)	49 (40.5%)	19 (25.7%)	18 (34.0%)	32 (41.0%)	31 (45.6%)		
**Diet**								
High risk	21 (13.8%)	15 (12.4%)	10 (13.5%)	5 (9.4%)	11 (14.1%)	19 (14.7%)	0.3493	0.883
Moderate risk	90 (59.2%)	72 (59.5%)	41 (55.4%)	31 (58.5%)	49 (62.8%)	41 (60.3%)		
Low risk	41 (27.0%)	34 (28.1%)	23 (31.1%)	17 (32.1%)	18 (23.1%)	17 (25.0%)		
Total score, M (SD)	5.6 (1.0)	5.8 (1.0)	5.5 (0.97)	5.6 (1.0)	5.8 (1.1)	5.9 (1.0)	–	–

a Statistics shown are *n* (valid %) unless otherwise specified.

b Unhealthy lifestyle behaviors were defined as follows. *High risk*: daily smoking, alcohol >9 W/>14 M drinks per week *and* binge drinking more than once a month, physical activity <75 min/week of vigorous exercise *and* <150 min low-intensity exercise/week, and diet score of 0–4 on the unhealthy diet index; *moderate risk*: intermittent smoking (not daily), alcohol >9 W/>14 M drinks per week *or* binge drinking more than once a month, physical activity <75 min/week of vigorous exercise *or* <150 min low-intensity exercise/week, and diet score of 5–8 on the unhealthy diet index. Additional information is available in Supplementary Table 1.

c The χ^2^ test was calculated by comparing the distribution of improved, unchanged, and worsened risk behaviors (3 categories) over the HP and TAU groups (2 categories) by time.

## Discussion

This study suggested it is possible to offer a digital self-help tool for healthy lifestyle promotion to primary care patients with mental health problems, who are receiving TAU. The overall recruitment rate was 21%, where improved recruitment routine at clinic B led to inclusion of around 40% of possible participants compared to 10% at clinic A. Women over 40 with moderate symptoms of depression and anxiety predominated among participating patients. Unhealthy lifestyle behaviors were present among 38% of the participants. Intervention adherence varied between clinics, where 70% of those randomized to the digital lifestyle intervention logged on to the platform at least once at clinic B, compared to 30% at clinic A. When MI counseling was available at clinic B, 29% opted to receive it. Results from the embedded recruitment trial showed that follow-up rates differed somewhat between groups: 88% in the TAU group and 73% in the intervention group. No between-group changes in depression, anxiety, or lifestyle behaviors occurred.

In sum, this study showed that it was possible to deliver a structured digital self-help intervention, with a focus on lifestyle behaviors, to patients who seek help for mental health problems in a primary care setting, when involving health professionals in the recruitment procedure, as in the second location. One striking result in this study was that the behaviors exhibited in this patient group did not seem particularly unhealthy, compared to the population in general. For instance, 5% in this study reported daily smoking compared to the 7% prevalence of daily smokers in the Swedish population (Makenzius, 2019). The smoking rate among study participants was surprisingly low since prior research indicates that mental health problems are associated with unhealthy lifestyle behaviors (Firth *et al.*, 2020). The relatively healthy behaviors in this study might be explained by the higher socioeconomic status of the participants; although such information was not collected in this study, comparison with national and regional data suggests that the inhabitants served by the clinics have better overall health and higher income than the general population (Makenzius, 2019; Statistics Sweden, 2021).

The findings raise the question of whether primary care patients seeking help for common mental health problems should be considered a risk group concerning unhealthy behaviors. It may be that the primary care sample in this study differs from the samples reported in the literature, in that the level of severe mental illness was lower in this sample. The recruitment rate was low at 21% and might improve should the intervention be offered only to patients with unhealthy lifestyle behaviors. An additional aspect is that it could be worthwhile to explore whether it is optimal to offer a lifestyle intervention when the patient has initially sought help, or whether it might be preferable to offer the intervention as a complement after patients complete treatment for mental health problems.

In this study, about one-third of the participants from clinic B opted to receive an MI counseling session. This session was offered in order to assess interest for an additional personal intervention, beyond the digital content of the Health Profile. An MI session is part of the original, paper-and-pencil version of the HP, and offering the intervention including MI was, as noted in the introduction, associated with healthy changes both in lifestyle and biometric outcomes (Blomstrand *et al.*, 2005; Blomstrand *et al.*, 2012). The MI session in the HP intervention has not previously been evaluated separately, and the finding that only one-third of the participants in this study chose an MI session suggests that it should remain optional. Motivational interviewing builds on an open-ended discourse on healthy behavior change (Berman *et al.*, 2020), and it is consistent with MI spirit to offer the session as an option. Indeed, a recent systematic review demonstrated that an open-ended conversation about behavior change is likely to reduce resistance to discussing healthy behavior changes (Albury *et al.*, 2019). However, primary care health professionals have expressed concerns about the risk of digital interventions leading to a deterioration of the relationship between health professionals and patients (Berman *et al.*, 2018). Complementing the HP with an optional MI session could be in line with recommendations that digital technology should serve to enhance the relationship between patient and clinician, not replace it (Torous and Roberts, 2017).

Regarding secondary outcomes from the embedded trial, no significant differences were found in short-term depression and anxiety outcomes between the group that received the digital lifestyle intervention with TAU and the TAU group, nor were there any significant differences in unhealthy behavior change between the two groups. The lack of significant effects for unhealthy behaviors may have been due to several factors. Patients were included whether they had unhealthy behaviors or not, and one important finding was that the majority of the patients did not exhibit any particularly unhealthy behaviors at baseline. Unhealthy behaviors in this group are of lower priority or could require more time.

### Future research

In a larger study, it could be relevant to conduct screening for unhealthy behaviors and to offer a lifestyle intervention only to patients with unhealthy behaviors. The findings that many patients did not log onto the platform, and that one-third opted to receive an MI session when offered, imply that there might also be a need to add more individualized and specific interventions for patients at risk for a specific unhealthy behavior. Such a change would align with current recommendations from the National Board of Health and Welfare, where ‘qualified counseling’ is the first-choice intervention and ‘simple advice’ is not prioritized (Socialstyrelsen, 2018). The format chosen to deliver the HP intervention was closer to a simple advice level of intervention, and increasing the personalization of the HP intervention could bring it closer to the qualified counseling level and thereby achieve a measurable level of effectiveness in healthy behavior changes.

It would also be of interest to further explore the prevalence of unhealthy behaviors in a socioeconomically broader primary care population seeking help for mental health problems, to gain a more in-depth understanding of whether this group really could be considered at risk for unhealthy behaviors, as prior research has suggested. Central requirements for carrying on with a larger RCT could be that a) there should be sufficient pace of recruitment at the clinic, b) participants should complete baseline and follow-up questionnaires to a large extent (approximately 80%), c) therapists should perceive the treatment as suitable for the clinical context, and d) patients should, to an acceptable extent, adhere to treatment. These requirements have largely been fulfilled in this study and could warrant a larger study with a sample restricted to patients with unhealthy lifestyle behaviors.

### Strengths and limitations

A strength of this study is that patients were recruited in the everyday setting of two different primary care clinics, implying that results are likely to generalize well to similar routine care contexts. Another strength is that there were few exclusion criteria, which means that the majority of primary care patients scheduled for in-house mental health assessment were eligible to participate. For example, there was no exclusion of patients reporting severe anxiety or depression symptoms, and there was no upper age limit. A strength was also the embedded recruitment trial, which provided preliminary data for a possible future RCT.

Limitations include the fact that additional information about the patients was not collected; e.g., socioeconomic variables and medication, because the study was planned not to interfere too much with routine care. A second limitation is that the inclusion rate was very slow in the first clinic and that the patient population may therefore not be representative. In addition, the LBQ lifestyle questionnaire, used in standard care in the region where the study was conducted, has not been formally validated although it is used as part of the digital medical record system in the Stockholm region where the study was conducted. Also, a drawback of the digital platform chosen was the limited possibility for systematically collecting information about usage of the intervention. Finally, the lack of long-term follow-up assessments could be construed as a limitation, but the value of long-term assessment is questionable given that the study was a pilot trial.

## Conclusion

In conclusion, it is possible to engage primary care patients seeking help for mental health problems in a digital intervention for promoting healthy lifestyle. In future research and/or implementation, it would seem crucial to offer the intervention only to patients with unhealthy lifestyle behaviors.

## Supplementary material

For supplementary material accompanying this paper visit http://doi.org/10.1017/S146342362300049X. click here to view supplementary material
