# Supplementary material for: Healthy lifestyle promotion via digital self-help for mental health patients in primary care: a pilot study including an embedded randomized recruitment trial
Source: Prim Health Care Res Dev. 2023 Sep 20;24:e56. doi: 10.1017/S146342362300049X (PMC10539741; doi:10.1017/S146342362300049X)
Supplement: Supplementary file 1 [file S146342362300049Xsup001.docx]

Supplement 1.

**Lifestyle behavior questionnaire (LBQ)**

**Tobacco consumption**

*Mark one alternative for each question – your usual behavior*

1. **My smoking habits - tobacco**
   - I have never been a smoker
   - I have been a smoker but have quit:

date of smoking cessation ………….

- - I quit smoking more than 6 months ago
  - I smoke, but not daily
  - I smoke daily… cigarettes/day
  - I smoke tobacco other than cigarettes daily

1. **My snuff habits – snuff boxes**
   - I have never used snuff regularly
   - I have used snuff regularly but have quit:

date of snuffing cessation ………….

- - I quit snuffing more than 6 months ago
  - I use snuff, but not daily.
  - I use snuff daily… boxes/week

2/3

**Alcohol Habits**

Definition of standard drink: one ”standard drink” means 50 cl medium-strong beer, 33 cl strong beer, 12-14cl white or red wine, 8 cl fortified wine, 4 cl spirits for instance whiskey

1. **How many standard drinks do you drink in a typical week?**

Drink ….. standard drinks per week

1. **How often do you drink 5 standard drinks or more on one occasion?**

.…. times per month

**Physical activity**

1. **How much time do you spend in a typical week on physical exercise that makes you short of breath, such as running, gymnastics, ball sports?**

….. minutes per week

1. **How much time do you spend in a typical week on everyday exercise, for example walking, cycling, gardening? Add up all time (at least 10 minutes at a time)**

….. minutes per week

**Eating habits**

*Mark one option for each question – what you usually do!*

1. **How often do you eat vegetables and/or root vegetables (fresh, frozen or cooked)?**
   - Twice per day or more often
   - Once per day
   - A few times a week
   - Once a week or less often
2. **How often do you eat fruit and/or berries (fresh, frozen, canned, juice, etc.)?**
   - Twice per day or more often
   - Once per day
   - A few times a week
   - Once a week or less often
3. **How often do you eat fish or seafood as a main course?**
   - Three times a week or more often
   - Twice a week
   - Once a week
   - A few times a month or less often
4. **How often do you eat pastry, chocolate/sweets, or soft drinks/juice?**
   - Twice per day or more often
   - Once per day
   - A few times a week
   - Once a week or less often
5. **How often do you eat breakfast**
   - Daily
   - Almost every day
   - A few times a week
   - Once a week or more

**Analysis procedure**

Response options for smoking are: daily use (with number of doses), non-daily use, previous but not current use (with date of cessation), and non-use. Questions about alcohol cover standard drinks per week and the frequency of binge drinking. The physical activity questions cover time devoted to vigorous exercise and everyday exercise like walking, biking, or gardening. Finally, questions about diet cover the consumption of vegetables, fruits, sweets, fish and seafood, resulting in a *diet index* scored from 0 to 14; <5 being indicative of “significantly unhealthy diet”, 5-8 “partly following guidelines”, and 9-14 “following guidelines”. For the total unhealthy Lifestyle Behavior score we constructed an index score for each area, ranging from 0 = high risk, 1 = moderate risk, 2 = low risk. The total unhealthy lifestyle behavior score thus ranges from 0-8, where lower scores indicate more unhealthy behaviors, and higher scores correspondingly indicate healthier behaviors.

| Smoking | 0  Daily smoker | 1  Smoker, but not daily | 2  Never been a smoker or quit smoking |
| --- | --- | --- | --- |
| Alcohol | 0  Drinking over 9/14 (W/M) standard drinks/week AND binge drinking more often than once/month | 1  Drinking over 9/14 (W/M) standard drinks/week OR binge drinking more often than once/month | 2  Drinking 9/14 (W/M) or fewer standard drinks/week AND binge drinking no more than 4/5 standard drinks once/month |
| Physical Activity | 0  Insufficient physical activity. Less than 75 minutes of vigorous exercise AND 150 minutes of weekly exercise | 1  Insufficient physical activity. Less than 75 minutes of vigorous exercise OR 150 minutes of weekly exercise | 2  Sufficient physical activity. Minimum of 75 minutes of vigorous exercise AND 150 minutes of weekly exercise |
| Diet | 0  0-4 scores on the unhealthy diet index “significantly unhealthy diet” | 1  5-8 scores  “partly following guidelines” | 2  9-12 scores “following guidelines” |
